# Over 40 years (1981–2023) assessing stigma with the Community Attitudes to Mental Illness (CAMI) scale: a systematic review of its psychometric properties

**DOI:** 10.1186/s13643-023-02230-4

**Published:** 2023-04-14

**Authors:** Juan P. Sanabria-Mazo, Eduardo Doval, Albert Bernadàs, Natalia Angarita-Osorio, Ariadna Colomer-Carbonell, Sara Evans-Lacko, Graham Thornicroft, Juan V. Luciano, María Rubio-Valera

**Affiliations:** 1 Teaching, Research & Innovation Unit, Parc Sanitari Sant Joan de Déu, Catalonia, Spain; 2 Centre for Biomedical Research in Epidemiology and Public Health (CIBERESP), Madrid, Spain; 3 Department of Basics, Developmental and Educational Psychology, Autonomous University of Barcelona, Barcelona, Spain; 4 Department of Psychobiology and Methodology of Health Sciences, Autonomous University of Barcelona, Barcelona, Spain; 5 Mental Health Research Group, Hospital del Mar Medical Research Institute (IMIM), Barcelona, Spain; 6 Care Policy and Evaluation Centre, London School of Economics and Political Science, London, UK; 7 Centre for Global Mental Health and Centre for Implementation Science, Institute of Psychiatry, Psychology & Neuroscience, Kin’s College London, London, UK; 8 Department of Clinical and Health Psychology, Autonomous University of Barcelona, Barcelona, Spain

**Keywords:** Stigma, Mental health, Community attitudes to mental illness, Psychometric properties, Systematic review

## Abstract

**Background:**

The Community Attitudes to Mental Illness (CAMI) scale measures social stigma towards people with mental illness. Although it has been used worldwide, the psychometric properties of the CAMI have not been systematically reviewed. The main aim of this study was to systematically review the psychometric properties of the different versions of the CAMI more than 40 years after of its publication.

**Methods:**

A systematic search was conducted in MEDLINE, PsycINFO, Web of Science, and EMBASE from 1981 (year of publication) to 2023 (present). A double review was performed for eligibility, data extraction, and quality assessment.

**Results:**

A total of 15 studies enrolling 10,841 participants were included. The most frequently reported factor structure comprises 3 or 4 factors. Overall, the internal consistency seems adequate for the global scale (*α* ≥ 0.80), except for CAMI-10 (*α* = 0.69). Internal consistency of the subscales are not supported, with authoritarianism being the weakest factor (*α* = 0.27 to 0.68). The stability over time of the total scale has been assessed in the CAMI-40, CAMI-BR, and CAMI-10 (*r* ≥ 0.39). Few studies have assessed the temporal stability of the CAMI subscales. Most of the correlations with potentially related measures are significant and in the expected direction.

**Conclusions:**

The 3 and 4 factor structure are the most widely reported in the different versions of the CAMI. Even though reliability and construct validity are acceptable, further item refinement by international consensus seems warranted more than 40 years after the original publication.

**Systematic review registration:**

PROSPERO identification number: CRD42018098956.

**Supplementary Information:**

The online version contains supplementary material available at 10.1186/s13643-023-02230-4.

## Introduction

Stigma towards people with mental disorders is a sociocultural phenomenon [1, 2] that negatively affects quality of life, self-esteem, interpersonal relationships, health care seeking and provision, and workplace integration [3, 4]. In Europe, it is estimated that there are about 165 million people with mental disorders, and it is calculated that around 38% of people will experience a mental disorder in their lifetime [5]. A recent systematic review confirmed that mental disorders cause a substantial economic burden for societies, with developmental disorders, schizophrenia, and intellectual disabilities obtaining the top median societal cost per patient [6].

Although there is not a universally accepted definition of stigma, it can be considered a multidimensional construct composed of negative elements of knowledge, attitudes, feelings, beliefs, and behaviours towards a group of people [7]. Stigma is a powerful barrier to social participation and professional help-seeking for people with mental disorders and there is a widespread social belief that such people are aggressive and uncontrollable [8–12]. In the last decade, various countries have implemented antidiscrimination campaigns to reduce stigma and improve the integration of people with mental illness into communities [13]. Examples of such programmes are *Time to Change* in England [14]; *Obertament* in Spain [15]; *Schizophrenia has many faces* in Austria [16]; *Like minds like mine* in New Zealand [17]; *One of us* in Denmark [18]; and *Opening minds* in Canada [19].

There is a long history of scales developed to assess attitudes towards mental illness. The *Opinion about Mental Illness* (OMI) [20] and the *Custodial Mental Illness Ideology Scale* (CMI) [21] were developed in the 1950s–1960s as the first scales to measure stigma. More recently, the *Community Mental Health Ideology* (CMHI) [22], the *Community Attitudes to Mental Illness Scale* (CAMI) [23], the *Mental Health Knowledge Schedule* (MAKS) [24], and the *California Assessment of Stigma Change* (CASC) [25] were designed. Since its publication in 1981, the CAMI [23] has been the gold standard measure for assessing stigma towards people with mental disorders. It has been translated into several languages (Spanish, Italian, Swedish, Portuguese, Greek, and Persian, among others) and used to measure stigma in a wide variety of samples (e.g., nurses, psychiatrists, and relatives of psychiatric patients).

The original version of the CAMI was partially derived from a brief, revised, and updated version of the OMI [20], and it was initially developed to predict the reactions of the general population to local services for people with severe mental disorders. This original version is composed of 40 items that are responded on a 5-point Likert scale, ranging from “strongly agree” to “strongly disagree”. According to its developers, the CAMI contains four subscales: authoritarianism, benevolence, social restrictiveness, and community mental health ideology [23]. Each subscale contains 10 items (5 positively formulated plus 5 negatively formulated) on the opinions of treating and caring for people with a serious mental disorder. Therefore, subscale scores can range from 10 to 50, with higher scores indicating less stigma towards people with mental disorders.

Given that the CAMI has been available for more than four decades, with hundreds of citations, the time is right for a systematic review of the psychometric properties of its different versions. As far as it is known, there are no previous reviews summarizing available psychometric information on the CAMI. This systematic review bridges this gap by synthesizing and critically appraising the psychometric properties of this stigma scale.

## Method

### Protocol and registration

This systematic review was conducted according to the Preferred Reporting Items for Systematic Reviews and Meta-analyses guidelines (PRISMA) [26]. The review protocol was registered in Prospective Register of Systematic Reviews (PROSPERO) on July 25th, 2018, under identification number: CRD42018098956.

### Search strategy

Searches were conducted through four electronic databases: MEDLINE (PubMed), PsycINFO (ProQuest), Web of Science (Core Collection), and EMBASE (Elsevier). The search strategy included terms related to psychometrics (psychometrics OR factor analysis OR reliability OR intra-class OR test–retest OR internal consistency OR validity OR dimensionality OR sensitivity to change OR responsiveness OR sensibility OR specificity) and to the original scale name ((attitude* AND toward* AND mental* AND Ill*) OR ("CAMI")), found using keywords in all fields and in the Medical Subject Headings (MeSH). The search string used in MEDLINE (PubMed) is shown in the Supplementary Table S1. Limits and filters were not activated in any of the database searches to avoid loss of potential eligible studies. The references of included studies were screened by reverse citation search to identify studies not detected in the electronic searches.

### Eligibility criteria

The search in the databases incorporated studies published in peer-reviewed journals from 1981 (when the original version was published [23]) to February 28th, 2023 (present). This systematic review included all studies that provided evidence on the psychometric properties (content validity, factor structure, internal consistency, test–retest reliability, construct validity, floor/ceiling effects, and interpretability) of the different versions of the CAMI. No restrictions were placed on the characteristics of the participants and the type of sampling used in the search. To ensure the rigour of the included studies, non-original studies (reviews, books, doctoral dissertations, commentaries, conference abstracts, study protocols, case reports, and qualitative studies, among others) and grey literature (i.e., non-peer-reviewed manuscripts) were excluded. Non-English, non-Spanish, or non-Italian papers were also excluded.

### Data management and study selection

In the first phase, duplicate articles in the databases were removed using Mendeley. In the second phase, two reviewers (AB and NA–O) independently assessed the articles based on their title and abstract according to the eligibility criteria. In the third phase, the full text of those articles that met the second phase was reviewed to verify compliance with the eligibility criteria. In the fourth phase, discrepancies in study selection were resolved with the help of two additional external reviewers (JVL and MR-V). In the fifth phase, relevant data were extracted from the selected documents with a standardised data extraction form and the respective quality assessment was carried out for each study.

### Data extraction

Data extraction from the selected articles was performed independently by two reviewers (AB and NA–O), using a template containing the following sections: authors, year of publication, country, CAMI version, study design, target population, sample type, sample size, age, gender, results depending on sociodemographic variables, and psychometric results about CAMI. The authors of the study were contacted to obtain additional information on the psychometric properties of the scale when it was necessary.

### Quality assessment

The quality of the included studies was assessed using the criteria proposed by Terwee et al. [27] for health measures. Each of the 7 criteria is scored 2 if the criteria are fulfilled, 1 if they are partially fulfilled, and 0 if no criteria are fulfilled. The total score can range from 0 to 14. The quality assessment was carried out by two reviewers (JPS-M and AB), with the supervision of two external reviewers (JVL and MR-V). Specifically, the following psychometric properties were assessed:

1. *Content validity* indicates whether the construct of interest is sampled by the questionnaire items. A score of 2 was assigned if the measurement objective of the questionnaire and the target population were explicitly defined. For this criterion to be met, it was necessary to develop the questionnaire items in consensus with the general population and stigma experts [27]. A score of 1 was given if some of the aspects mentioned above were missing and a score of 0 if none of the above information was described.
2. *Factor structure* refers to the dimensionality of the scale [27]. A score of 2 was given if an exploratory factor analysis (EFA) and a confirmatory factor analysis (CFA) had been performed on different samples or if the CFA had been calculated considering a theoretical model. This score was only given if the factor analyses supported the structure promoted by the authors. A score of 1 was awarded if only the EFA had been carried out, and if the EFA supported the factor structure. A score of 0 was awarded if factor analysis has not been conducted or if EFA or CFA does not support the proposed dimensionality.
3. *Internal consistency* is used to indicate the degree of reliability of a scale. For health scales, Cronbach’s *α* should be between 0.70 and 0.95 [27]. For greater rigour in the findings, the Nunnally and Bernstein [28] criteria were used in this study, which present scores above 0.80 as acceptable. A score of 2 was given if Cronbach's *α* was calculated for each dimension and if it was between 0.80 and 0.95. A score of 1 was assigned if internal consistence was calculated only for some dimensions and if it was below 0.80. A score of 0 was reported if no internal consistency information was found or if the evidence was questionable.
4. *Test–retest reliability* is a measure used to validate the stability of the scale over time. For acceptable temporal stability, the test–retest needs to be at least *r* = 0.70 [27, 29]. The intraclass correlation coefficient (ICC) is the most recommended statistical index for continuous measures in the assessment of temporal stability. To reduce possible recall bias, a score of 2 was indicated if the time interval between test administration was 1–2 weeks. A score of 1 was assigned if the time interval between test administration was less than 1 week or greater than 2 weeks and a score of 0 if no information on test–retest reliability was reported.
5. *Construct validity* addresses whether scores on a questionnaire are significantly associated with potentially related measures. A theoretical underpinning is needed to verify the hypotheses of expected correlations between different scales. At least two of the correlations between two theoretically related constructs had to have a minimum of *r* = 0.50 [27]. A score of 2 was given if information about convergent validity and divergent validity was provided. A score of 1 was assigned if information was provided for only one of the concepts mentioned above and score of 0 if no construct validity information was provided.
6. *Floor and ceiling effects* is a measure to detect the number of participants achieving the highest or lowest possible scores [27]. A score of 2 was assigned if less than 15% of respondents achieved the highest or lowest possible scores. A score of 1 was reported if more than 15% of respondents achieved the highest or lowest possible scores and a score of 0 if this information was not provided.
7. *Interpretability* indicates how differences in scores on the CAMI can be interpreted or the degree to which qualitative meaning can be obtained from quantitative scores. A known-groups validity approach is suggested with means and standard deviations (SDs) of scores of relevant subgroups of participants who are expected to differ in the CAMI [27]. A score of 2 was assigned if mean and SDs of four or more relevant groups were reported. A score of 1 was given if mean and SDs of less than four relevant groups were informed and score of 0 if no information about interpretability was found.

## Results

### Selection and inclusion of studies

**Fig. 1:**
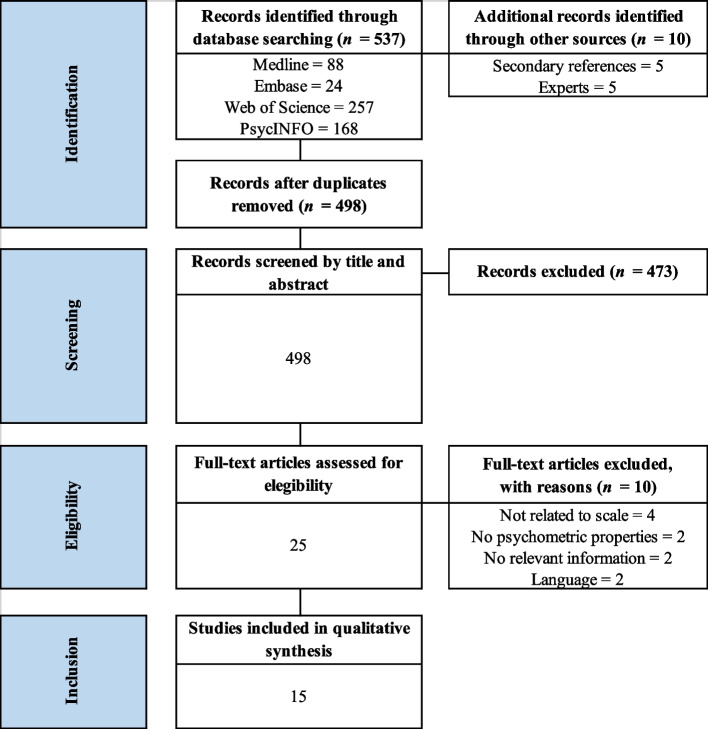
PRISMA flowchart from record identification to study inclusion

As displayed in Fig. 1, the initial database search yielded a total of 537published articles. In addition, 5 articles were included by reverse citation and 5 by experts. After removal of duplicates, 498 titles and abstracts were reviewed, of which 25 was selected for full-text review. After this process, 10 articles were excluded, 4 articles because they were not related to the CAMI, 2 because they did not focus on the psychometric properties of the scale, 2 because they did not provide relevant information, and 2 because it was written in other languages (German or Chinese). Finally, a total of 15 studies were included in this systematic review.

### Characteristics of included studies

The 15 included studies were conducted in 12 different countries: Spain (*n* = 2), United Kingdom (*n* = 2), Italy (*n* = 1), France (*n* = 1), Sweden (*n* = 1), Ireland (*n* = 1), Canada (*n* = 1), China (*n* = 1), Chile (*n* = 1), Argentina (*n* = 1), Kenya (*n* = 1), and Iran (*n* = 1). Participants in most studies were healthy individuals (*n* = 13) and the most frequent type of sampling was non-probability (*n* = 12). The sample size of the studies ranged from 130 [30] to 2000 [31], enrolling a total of 10,841 participants, and the mean age ranged from 15 [32] to 48 [15] years old. The proportion of women in all studies was higher than 50%. The design of all included studies was observational.

The included studies were published between 1981 [23] and 2023 [30]. The original CAMI-40 (*n* = 5) [23, 30, 32–34] and the CAMI-20 (*n* = 3) [33, 35, 36] were the most psychometrically analysed versions, followed by CAMI-W (*n* = 2) [33, 37], CAMI-26 (*n* = 2) [15, 38], CAMI-BR (*n* = 1) [39], CAMI-31 (*n* = 1) [31], CAMI-10 (*n* = 1) [40], CAMI-24 (*n* = 1) [41], CAMI-22 (*n* = 1) [42], CAMI-1 [33], CAMI-2 [33], and CAMI-W (*n* = 1) [33]. In total, 14 studies examined the psychometric properties of CAMI using classical test theory [15, 23, 30–33, 35–42] and one using item response theory (IRT) [34]. Table 1 provides a detailed description of the included studies.

**Table 1:** Characteristics of the studies included in the systematic review (*n* = 13)

Author (year). Country	CAMI version (items)	Target population and study design (sample type)	Sample size. Mean age (SD). % female	Results depending on sociodemographic variables	Psychometric results about CAMI
Taylor and Dear (1981) [23]. Canada	CAMI-40 (40 items)	• General population from Toronto with separate samples from areas with and without mental health facilities • Observational (non-probabilistic sample)	• 1090 participants • Mean age: NA (NA). Female: NA	• Less considerate attitudes toward the mentally ill: elder people, divorced people, users with children (not including the ones with children older than 18 years old), regular church attenders (depending on the religious determination). The Pentecostal and Greek Orthodox groups showed more authoritarian attitudes and the Pentecostal and Greek Orthodox had the least benevolent attitudes • More considerate attitudes toward the mentally ill: female respondents, higher sociodemographic status, and higher education. The users that had used mental health facilities or are related with friends that had used those facilities. The Baptists and Salvation Army showed fewer authoritarian attitudes and the Baptists with the United Church, showed more benevolent attitudes	Factor structure • Benevolence (10 items) • CMHI (10 items) • Social restrictiveness (10 items) • Authoritarianism (10 items) Internal consistency • Global (NA) • Subscales: benevolence (*α* = 0.76), CMHI (*α* = 0.88), social restrictiveness (*α* = 0.80), and authoritarianism (*α* = 0.68) Test–retest reliability • Global (NA) • Subscales (NA) Validity • A strong degree of correspondence between the factor scales and the theoretically ones. It is important to emphasize that the authoritarianism and social restrictiveness scales equally correlated with the first factor, ad to a lesser extent, with the fourth factor
Brockington et al. (1993) [31]. United Kingdom	CAMI-31 (31 items)	• General population from the Malvern and Bromsgrove area • Observational (non-probabilistic sample)	• 2000 participants • Bromsgrove mean age: NA (NA). Female: 51% • Malvern mean age: NA (NA). Female: 58%	• CMHI: (factor 1). Higher education and professional experience with people with mental health problems • Authoritarianism (factor 2). Lower scores are related with higher education • Benevolence (factor 3). Highest scores were found within the groups with higher education, knowledge of mental illness, and an age between 55 and 64	Factor structure • Benevolence (NA) • CMHI (NA) • Authoritarianism (NA) Internal consistency • Global (NA) • Subscales (NA) Test–retest reliability • Global (NA) • Subscales (NA) Validity • NA
Wolff et al. (1996) [37]. United Kingdom	CAMI-W (20 items)	• General population from London • Observational (non-probabilistic sample)	• 215 participants • Mean age: 37 (13). Female: 55%	• Fear and exclusion were associated with having children in the domestic establishment (< 18), higher age, and a lower occupational status • Social control was associated with a higher age; sociodemographic lower class; Asian, African, or Caribbean descendant; lower educational level; with children in the household; lower occupational status; being divorced, widowed, or separated; and be aware of somebody with a mental health problem • Goodwill was associated with lower age, higher educational level, and people from ethnical origin	Factor structure • Fear and exclusion (11 items) • Social control (6 items) • Goodwill (3 items) Internal consistency • Global (NA) • Subscales (NA) Test–retest reliability • Global (NA) • Subscales (NA) Validity • Predictive validity. Significant correlation between fear and exclusion, social control and goodwill, and behavioural intention items
Song et al. (2005) [42]. China	CAMI-22 (22 items)	• General population from Taiwan • Observational (stratified proportional sample)	• 1203 participants • Mean age: 42 (14). Female: 51%	• NA	Factor structure • Benevolence (6 items) • Rehabilitation in the community (6 items) • Non-authoritarism (5 items) • Non-social restrictiveness (3 items) • Normalization (2 items) Internal consistency • Global (NA) • Subscale: benevolence (*α* = 0.63), rehabilitation in the community (*α* = 0.61), non-authoritarism (*α* = 0.52), non-social restrictiveness (*α* = 0.53), and normalization (*α* = 0.43) Test–retest reliability • Global (NA) • Subscale (NA) Validity • NA
Buizza et al. (2005) [41]. Italy	CAMI-24 (24 items)	• General population from Brescia • Observational (probabilistic sample)	• 280 participants • Mean age: 48 (NA). Female: 60%	• Physical distance and fear. They are associated with age > 61 years, being divorced/widowed/separated, and never having participated in voluntary or social activities • Social isolation. Associated with age > 41 years, higher education, and being unemployed • Social responsibility and tolerance. There are no significative association between the social demographic variables and this factor	Factor structure • Physical distance and fear (8 items) • Social distance and isolation (9 items) • Social responsibility and tolerance (7 items) Internal consistency • Global (NA) • Subscales (NA) Test–retest reliability • Global (NA) • Subscales (NA) Validity • NA
Högberg et al. (2008) [35]. Sweden	CAMI-20 (20 items)	• Student nurses selected based on their easy availability • Observational (non-probabilistic sample)	• 421 participants • Mean age: 28 (7). Female: NA	• NA	Factor structure • Open-minded and pro-integration (9 items) • Fear and avoidance (6 items) • Community mental health ideology (5 items) Internal consistency • Global (α = 0.90) • Subscales (NA) Test–retest reliability • Global (NA) • Subscales (NA) Validity • NA
Morris et al. (2011) [33]. Ireland	CAMI-40 (40 items), re-specified CAMI-1 (NA), re-specified CAMI-2 (NA), Högberg CAMI-20 (20 items), CAMI-W (20 items), Re-specified Wolff Scale (NA)	• Nurses • Observational (non-probabilistic sample)	• 1242 participants • Mean age: 40 (10). Female: 66%	• NA	Factor structure CAMI-40 • Authoritarianism (10 items) • Benevolence (10 items) • Social restrictiveness (10 items) • CMHI (10 items) Re-specified CAMI 1 • NA Re-specified CAMI 2 • NA Högberg CAMI-20 • Open-minded and pro-integration (9 items) • Fear and avoidance (6 items) • Community mental health ideology (5 items) Wolff CAMI-20 • Fear and exclusion (11 items) • Social control (6 items) • Goodwill (3 items) Re-specified Wolff scale • NA Internal consistency • Global (NA) • Subscales (NA) Test–retest reliability • Global (NA) • Subscales (NA) Validity • NA
Abelha et al. (2015) [39]. Brazil	CAMI-BR (40 items)	• General population from the Rio de Janeiro area • Observational (non-probabilistic sample)	• 230 participants • Mean age: 45 (3). Female: 68%	• NA	Factor structure • Social restrictiveness (10 items) • Benevolence (10 items) • CMHI (10 items) • Authoritarism (10 items) Internal consistency • Global (*α* = 0.84) • Subscales: social restrictiveness (*α* = 0.76), benevolence (*α* = 0.69), CMHI (*α* = 0.81), and authoritarianism (*α* = 0.35) Test–retest reliability • Global (*r* = 0.69) • Subscales: social restrictiveness (*r* = 0.64), benevolence (*r* = 0.62), CMHI (*r* = 0.54), and authoritarianism (*r* = 0.37) Validity • NA
Ochoa et al. (2016) [32]. Spain	CAMI-40 (40 items)	• Elementary school students • Observational (non-probabilistic sample)	• 150 participants • Mean age: 15 (1). Female: 51%	• NA	Factor structure • Authoritarianism (10 items) • Benevolence (10 items) • Social restrictiveness (10 items) • CMHI (10 items) Internal consistency • Global: first assessment (*α* = 0.86) and second assessment (*α* = 0.90) ^a^Data provided by Ochoa et al. [34] • Subscales: authoritarianism (*α* = 0.27), benevolence (*α* = 0.64), social restrictiveness (*α* = 0.67), and CMHI (*α* = 0.81) Test–retest reliability • Global (NA) • Subscales: CMHI (*r* = 0.88), authoritarianism (*r* = 0.81), benevolence (*r* = 0.85), and social restrictiveness (*r* = 0.81) Validity • NA
Grandón et al. (2016) [40]. Chile	CAMI-10 (10 items)	• Two samples of the general population from Concepción • Observational (non-probabilistic simple)	• 749 participants form pooling 2 samples • First sample: mean age: 39 (13). Female: 56% • Second sample: mean age: 42 (13). Female: 56%	NA	Factor structure • Acceptance (5 items) • CMHI (5 items) Internal consistency • Global (*α* = 0.69) Subscales: acceptance (*α* = 0.61) and CMHI (*α* = 0.66) Test–retest reliability • Global (*r* = 0.79) • Subscales: CMHI (*r* = 0.63), authoritarianism (*r* = 0.57), benevolence (*r* = 0.39), and social restrictiveness (*r* = 0.62) Validity • Factor 1 SDO-factor 1 CAMI (− 0.25) • Factor 1 SDO–factor 2 CAMI (− 0.30) • Factor 2 SDO–factor 1 CAMI (− 0.25) • Factor 2 SDO–factor 2 CAMI (0.16)
Rubio-Valera et al. (2016) [15]. Spain	CAMI-26 (26 items)	• General population • Observational (probabilistic sample)	• 1019 participants • Mean age: 48 (1). Female: 51%	• NA	Factor structure • Authoritarianism (7 items) • Benevolence (6 items) • Support for community mental health ideology (9 items) Internal consistency • Global (NA) • Subscales: authoritarism (*α* = 0.54), benevolence (*α* = 0.63), and support for community mental health care (*α* = 0.72) Test–retest reliability • Global (NA) • Subscales (NA) Validity • NA
García et al. (2017) [38]. France	CAMI-26 (26 items)	• Undergraduate nursing students • Observational (non-probabilistic)	• 268 participants • Mean age: NA (NA). Female: NA	• NA	Factor structure • Benevolence (7 items) • CMHI (5 items) • Authoritarism (7 items) • Restrictiveness (7 items) Internal consistency • Global (NA) • Subscales (NA) Test–retest reliability • Global (*r* = 0.79) • Subscales: CMHI (*r* = 0.63), authoritarianism (*r* = 0.57), benevolence (*r* = 0.39), and social restrictiveness (*r* = 0.62) Validity • CAMI is related to the housing control scale, RIBS, and MARKS
Tong et al. (2020) [36]. China	CAMI-20 (20 items)	• Medical students (MS) and primary healthcare workers (PHW) • Observational (non-probabilistic)	• 1228 MS and 1092 PGW • MS sample: mean age: 21 (2). Female: 63% • PHW sample: mean age: 36.3. Female: 70%	NA	Factor structure • Benevolence (5 items) • Fear and exclusion (8 items) • Support and tolerance (7 items) Internal consistency • Global: MS (α = 0.82) and PHW (α = 0.85) • Subscales: MS and PHW, respectively: benevolence (α = 0.79 and 0.83), fear and exclusion (α = 0.79 and 0.85), support and tolerance (α = 0.74 and 0.85) Test–retest reliability • Global: MS (*r* = 0.79) and PHW (*r* = 0.75) • Items: MS (*r* = 0.29 to 0.61) and PHW (*r* = 0.45 to 0.74) Validity • Convergent validity. Correlation with potentially related measures was not assessed • Discriminant validity. Good discriminant validity between subgroups of interest (MS and PHW)
Bitta et al. (2022) [34]. Kenya	CAMI-40 (40 items)	• Population from the Kilifi County Hospital • Observational (non-probabilistic)	• 616 participants • Mean age: 37 (14). Female: 51%	• NA	Factor structure • Authoritarianism (13 items) • Benevolence (7 items) • CMHI (3 items) Internal consistency • Global (*ω* = 0.78) • Subscales (NA) Test–retest reliability • Global (*r* = 0.39) • Subscales (NA) Validity • RIBS-factor 1 CAMI (− 0.26) • RIBS-factor 2 CAMI (0.13) • RIBS-factor 3 CAMI (0.31) • RIBS-CAMI total (− 0.08) • MAKS total-factor 1 CAMI (− 0.10) • MAKS total-factor 2 CAMI (0.16) • MAKS total-factor 3 CAMI (0.34) • MAKS total-CAMI total (0.09) Item responses • People with mental illness experiences reported significantly better attitudes than those without • Women had significantly higher scores in factor one scores compared to men • Level of education had a significant association with overall scores for factor one, but not factor two or three
Kafami et al. (2023) [30]. Iran	CAMI-40 (40 items)	• General population • Observational (non-probabilistic)	• 130 participants • Mean age: 35 (11). Female: 79%	• NA	Factor structure NA Internal consistency • Global (α = 0.59) • Subscales: authoritarianism (α = 0.61), benevolence (α = 0.49), social restrictiveness (α = 0.64), and CMHI (α = 0.76) Test–retest reliability • Global (*r* = 0.93) • Subscales: authoritarianism (*r* = 0.97), benevolence (*r* = 0.92), social restrictiveness (*r* = 0.95), and CMHI (*r* = 0.95) Validity • NA

^a^In those psychometric studies where a factor analysis has been carried out, resulting, for the most part, in several different factors and items, it has been decided, in the CAMI section used, to indicate the version resulting from the factor analysis previously carried out. Brockington et al. [38] – Not all the items are indicated due to their non-specification in the original article

### Quality assessment

As shown in Table 2, the overall methodological quality of the included studies was low. On a scale of 0 to 14 points, 8 studies scored 5 or less [30, 31, 33, 35, 37, 38, 41, 42] and 6 scored 6–7 [15, 23, 32, 34, 39, 40]. Only one study [36] scored higher than 10 on the quality assessment, which indicates that in general the psychometric properties of the CAMI have not been adequately assessed.

**Table 2:** Quality assessment of the included studies using the criteria proposed by Terwee et al. [27]

Articles	Content validity	Factor structure	Internal consistency	Test–retest reliability	Construct validity	Floor/ceiling effects	Interpretability	Final score (0–14)
Taylor and Dear (1981) [23]	2	1	1	0	1	0	1	6
Brockington et al. (1993) [31]	1	1	0	0	0	0	1	3
Wolff et al. (1996) [37]	2	1	0	0	0	0	2	5
Song et al. (2005) [42]	2	1	0	0	0	0	1	4
Buizza et al. (2005) [41]	2	1	0	0	0	0	2	5
Högberg et al. (2008) [35]	2	1	1	0	0	0	0	4
Morris et al. (2011) [33]	2	2	1	0	0	0	0	5
Abelha et al. (2015) [39]	2	1	1	0	1	0	1	6
Ochoa et al. (2016) [32]	2	0	1	2	0	0	1	6
Grandón et al. (2016) [40]	2	2	1	0	1	0	1	7
Rubio et al. (2016) [15]	2	1	1	0	0	0	2	6
García et al. (2017) [38]	2	1	0	1	1	0	0	5
Tong et al. (2020) [36]	2	2	2	2	1	0	2	11
Bitta et al. (2022) [34]	1	1	1	1	1	0	1	6
Kafami et al. (2023) [30]	2	0	1	1	0	0	0	4

The quality of the included studies was assessed using the criteria proposed by Terwee et al. [27] for health measures. Each of the criteria is scored 2 if the criteria are fulfilled, 1 if they are partially fulfilled, and 0 if no criteria are fulfilled. The total score can range from 0 to 14

### Content validity

The construct of interest in all included studies (*n* = 15) was the assessment of attitudes towards people suffering from mental disorders. The developers of the CAMI generated part of the scale by extracting items from previously published measures [23], whereas the subsequent versions were adaptations with different length of the original scale.

### Factor structure

The dimensionality of the CAMI was assessed in most of the included articles (*n* = 11). Three studies computed a CFA [34, 35, 38], six opted for an EFA or principal component analysis [15, 23, 31, 37, 40, 42], and 2 computed EFA and CFA [38, 39]. The number of dimensions ranged between 2 and 5. Regarding item allocation, it was found that items loaded on different subscales depending on the version of the CAMI. Considering suggested “Rules of thumb” [41, 43], in the original CAMI (40 items distributed in 4 factors) only 26 of the 40 items had a factor loading greater than 0.40, with a difference ≥ 0.15 in the factor loadings of each item in the different factors. In addition, some items load more strongly on other factors than on the original factor assignment reported by Taylor and Dear [23].

### Internal consistency

The internal consistency was assessed in 9 of the included studies. It was assessed for both the global CAMI (*n* = 7) [23, 30, 32, 34, 35, 39, 40] and its subscales (*n* = 7) [15, 23, 30, 32, 39, 40, 42]. All studies that assessed the Cronbach’s α or McDonald`s Omega (*ω*) of the global scale, both original CAMI-40 and its versions (i.e., CAMI-BR, CAMI-20, and CAMI-10), exceeded the minimum established by Nunnally and Bernstein [28] of 0.80, except for three studies: (*ω* = 0.78 for the CAMI-40) [34], (α = 0.59 for the CAMI-40) [30], and (α = 0.69 for the CAMI-10) [40]. Studies that assessed the Cronbach’s α of the subscales in the original CAMI-40 [23, 30, 32] and its versions (i.e., CAMI-26, CAMI-BR, CAMI-10, and CAMI-22) [15, 37, 39, 40] showed some heterogeneity in their coefficients. Of these, some (*n* = 4) [15, 30, 40, 42] reported internal consistency values below the recommended minimum cut-off point in the CAMI subscales.

On one hand, the study assessing the subscales of the CAMI-22 [42] reported internal consistency values below the minimum recommended score on benevolence (*α* = 0.63), non-authoritarianism (*α* = 0.52), non-social restrictiveness (*α* = 0.53), normalisation (*α* = 0.43), and community rehabilitation (*α* = 0.61). The study examining the subscales of the CAMI-10 [40] obtained alpha values below the minimum recommended cut-off point on acceptance (all *α* < 0.70). The study assessing the subscales of the CAMI-26 [15] reported alpha values below the recommended cut-off point on benevolence (*α* = 0.63), authoritarianism (*α* = 0.54), and support for the mental health community (*α* = 0.72).

On the other hand, there were studies that reported a Cronbach’s α value below the recommended cut-off only in some specific subscales of the original CAMI-40 [23, 30, 32] and the CAMI-BR [39]. The three studies [23, 30, 32] that evaluated the original CAMI indicated values below the cut-off on the dimensions of benevolence (*α* = 0.64 [23]; *α* = 0.76 [32]; *α* = 0.49 [30]), authoritarism (*α* = 0.27 [23]; *α* = 0.68 [32]; *α* = 0.61 [30]), social restrictiveness (α = 0.67 [32]; *α* = 0.64 [30]), and CMHI (*α* = 0.76 [30]). The study [39] focused on the subscales of the CAMI-BR reported very low alpha values on benevolence (*α* = 0.69), authoritarism (*α* = 0.35). In general, authoritarism is the least reliable subscale [23, 32, 39, 40, 42].

Regarding the original CAMI, two [23, 32] out of three studies that assessed its subscales reported an acceptable internal consistency on the CMHI subscale (*α* = 0.88; *α* = 0.81, respectively), while only one [23] of the two studies reported an adequate value in social restrictiveness (*α* = 0.80). The study [39] that assessed the reliability of the CAMI-BR subscales also reported an adequate value on the CMHI subscale (*α* = 0.81).

### Test–retest reliability

The temporal stability was assessed in five studies [30, 32, 34, 38, 39] by computing the ICC coefficient. One study that used the original CAMI [32] calculated temporal stability on all subscales, without reporting the global coefficient: benevolence (*r* = 0.85), authoritarianism (*r* = 0.81), social restrictiveness (*r* = 0.81), and CMHI (*r* = 0.88). Other study [34] explored the temporal stability of the original CAMI in the global coefficient (*r* = 0.39), with questionable results, but not in its subscales. The study that evaluated the temporal stability of the original CAMI [30] reported excellent results in both the global coefficient (*r* = 0.93) and its subscales: authoritarianism (*r* = 0.97), benevolence (*r* = 0.92), social restrictiveness (*r* = 0.95), and CMHI (*r* = 0.95).

The study assessing temporal stability on the CAMI-BR [39] found acceptable temporal stability for the overall scale (*r* = 0.69). However, the subscales of benevolence (*r* = 0.62), authoritarianism (*r* = 0.37), social restrictiveness (*r* = 0.64), and CMHI (*r* = 0.54) did not exhibit good temporal stability. The temporal stability of the CAMI-10 [40] was acceptable only for the overall scale (*r* = 0.79), but not in the case of the subscales: benevolence (*r* = 0.39), authoritarianism (*r* = 0.57), social restrictiveness (*r* = 0.62), and CMHI (*r* = 0.63).

### Construct validity

Three studies found positive statistically significant correlations of low magnitude (< 0.50) between the CAMI and potentially related instruments such as the MAKS and the Reported and Intended Behaviours Scale (RIBS). One study [40] identified correlations between the factors of the CAMI-10 (i.e., CMHI, authoritarianism, benevolence, and social restrictiveness) and the Social Dominance Orientation (SDO), with values ranging from − 0.31 to 0.16. Other study [38] found negative correlations between the CAMI-26 and instruments such as the RIBS (− 0.44) and the MAKS (− 0.30). Finally, one study [34] identified a negative correlation between original CAMI and RIBS (− 0.08) and a positive correlation with MAKS (0.09).

### Floor and ceiling effects

None of the included studies reported information on ceiling and/or floor effects.

### Interpretability

The interpretability was analysed from a known-groups validity approach in several studies (*n* = 11) [15, 23, 31, 32, 36–42]. For instance, older participants with low employment status and low social class have higher CAMI scores compared to younger participants and those with high employment and social status [31, 33]. Men also reported higher scores than women [15]. As expected, those participants who had undertaken volunteering or social activities scored lower than those who had not [41].

## Discussion

### Principal findings and interpretation

The results of this systematic review can be summarized as follows. The CAMI has been used in a wide variety of settings and in diverse samples from many different countries (i.e., Spain, United Kingdom, Italy, France, Sweden, Ireland, Canada, China, Chile, Argentina, Kenya, and Iran). The target population of included studies varied from students [32] and primary healthcare workers [33, 36] to the general population [15, 30, 31, 34, 37, 38, 40–42]. These aspects, besides the different CAMI versions used, might account for the heterogeneous findings in the psychometric data of this stigma measure.

In the current systematic review, a total of 15 papers met the inclusion criteria and provided data on several psychometric indices. Although the 3-factor model was the most reported structure [15, 31, 34, 35, 37, 41] followed by a 4-factor model [23, 38, 39], the items considerably varied in their expected allocation among studies. Only 3 out of 15 studies presented the same number of items [23, 30, 32].

There were three studies using the 40-item version that differed from the original dimensions proposed for the CAMI. Two studies [15, 34] supported a 3-factor structure, whereas the other supported a 4-factor structure [38]. The studies with more dramatic changes in item allocation were those using the CAMI-24 [41], CAMI-10 [40], and CAMI-26 [15]. The estimation method of maximum likelihood can only be used in CFA when multivariate normality is met, but in the CAMI-24 study [41] this assumption was violated. Moreover, the different approaches to analyse dimensionality (principal component analysis, EFA, and CFA) and the diverse methods used to estimate the factor models might explain the heterogeneous findings in dimensionality.

Regarding the internal consistency of the CAMI, 9 out of 15 studies addressed this psychometric aspect. Unexpectedly, 7 studies reported the α or *ω* for the total scale, whereas 7 reported α values only for subscales. According to Nunnally and Bernstein criteria [28], those studies that assessed the internal consistency of the total scale (CAMI-BR [39], CAMI-40 [32], and CAMI-20 [35]) obtained adequate alpha values, except for two studies with CAMI-40 [22, 34] and one study with the CAMI-10 [40]. In contrast, the studies that computed Cronbach’s α of each subscale [15, 23, 30, 32, 39, 40, 42], usually obtained values below the recommended cut-off of 0.80. More specifically, the authoritarism subscale was the least reliable subscale [23, 30, 32, 39, 40, 42].

The CAMI-40 was the only version in which temporal stability of its subscales was analysed, showing adequate stability over time. CAMI-BR [39] and CAMI-10 [40] did not present good stability in the subscales but had an acceptable test–retest on the global scale. In general, there is a lack of longitudinal studies, therefore this psychometric aspect has not been exhaustively addressed.

The pattern of correlations between the CAMI and other potentially related constructs was statistically significant and in the expected directions, partially supporting the construct validity. Following Terwee et al. criteria [27], some correlations were not of the expected magnitude. Finally, the interpretability of the CAMI scale assessed by 11 studies highlight that young female participants who have undertaken volunteering activities and have high employment and/or social status, present less stigma towards people with mental disorders.

## Strengths and limitations

As far as it is known, this systematic review is the first to summarize the psychometric properties of the different versions of the CAMI since it was published 40 years ago. The CAMI has been administered to a wide variety of populations. Adapted criteria based on the consensus-based standards for the selection of health measurement instruments (COSMIN) were applied to evaluate the quality of the CAMI measurement properties and provided a comprehensive and qualitative synthesis of its current evidence. For transparency purposes, the review protocol was registered in PROSPERO, and an exhaustive search strategy was carried out, as well as a clear data extraction procedure. In contrast, the scope of this results might be considered as limited due to the exclusion of papers not written in English, Spanish, or Italian. The relatively few included studies in this systematic review underly the need of addressing with more emphasis some psychometric properties of this stigma measure, such as the factorial invariance across age, gender, or cultures.

## Conclusions

The CAMI psychometric properties have been examined mainly using classic test theory as a framework. This methodological approach does not allow an assessment of the quality of individual CAMI items and factors. Even though some evidence on its psychometric soundness is beginning to emerge from IRT, the evidence is limited at present. The study that explored the CAMI properties by means of IRT methods indicated significant item bias according to health status, gender, and education level. In general, people with mental health experiences, women, and people with lower levels of education scored higher on some of the three CAMI factors. IRT-based methods can provide valuable information for gauging the quality of individual CAMI items and response options across different levels of stigma. This methodology is also very useful for assessing differential item functioning according to sociodemographic variables of interest. In this sense, more evidence based on a IRT approach is needed for the different versions of the CAMI.

The 3- and 4-factor structure are the most widely reported in the different versions of the CAMI. The general lack of fit of the existing data to the four-factor scale originally proposed by Taylor and Dear (1981) [23] could be related to the diversity of CAMI versions explored and to the changes that have been introduced in the adaptations of this inventory during the last years. Additionally, it is frequent to find in the literature that modern psychometric approaches (i.e., based on CFA) fail to replicate the dimensionality of old instruments developed in the seventies or eighties, which were originally analysed with exploratory techniques [44]. Although this inventory has been employed in many samples from different cultures and with different languages, some aspects have been scarcely addressed. For example, time needed for completion, difficulties in understanding the items, or the scale’s acceptability have not been explored. As stated before, measurement invariance has not been assessed in more than 40 years of history. Additionally, as only a few of the included studies had a longitudinal design, it was not possible to draw firm conclusions about the temporal stability of the CAMI. In the limited evidence available, a lack of good test–retest reliability was detected. This low temporal stability could be related to factors such as social desirability or that stigma is not an enduring trait.

In the opinion of the team of researchers of this article, the next step should focus on item refinement to create a uniform set of items, especially considering the influence of culture and context on the expression of stigma. This task would imply collaboration among an international panel of stigma experts. The resulting candidate scale should be evaluated by using cognitive interviews and surveying different samples to reach a final version of the inventory with adequate dimensionality, reliability, and validity that would allow cross-cultural comparisons. In this sense, the development of tools with room for contextual adaptation could be a valuable scientific contribution for the future. Finally, considering the scientific interest that CAMI has generated for more than forty years, it is suggested that future systematic reviews update the available evidence every 10 years.

## Supplementary Information

**Additional file 1: Supplementary Table S1.** Detailed search strategy in PubMed (it was adapted to each database).

## Data Availability

All data included in this systematic review were extracted from published papers. The data used for the study are made available within the manuscript and supplementary materials.

## Abbreviations

CAMI: Community Attitudes to Mental Illness
CASC: California Assessment of Stigma Change
CMI: Custodial Mental Illness Ideology Scale
CMHI: Community Mental Health Ideology
COSMIN: Consensus-based standards for the selection of health measurement instruments
EFA: Exploratory factor analysis
CFA: Confirmatory factor analysis
MAKS: Mental Health Knowledge Schedule
MESH: Medical Subject Headings
OMI: Opinion about Mental Illness
PRISMA: Preferred Reporting Items for Systematic Reviews and Meta-analyses
PROSPERO: Prospective Register of Systematic Reviews
RIBS: Reported and Intended Behaviours Scale
SDO: Social Dominance Orientation
