# Supplementary material for: Over 40 years (1981–2023) assessing stigma with the Community Attitudes to Mental Illness (CAMI) scale: a systematic review of its psychometric properties
Source: Syst Rev. 2023 Apr 14;12:66. doi: 10.1186/s13643-023-02230-4 (PMC10103533; doi:10.1186/s13643-023-02230-4)
Supplement: Supplementary file 1 — Additional file 1: Supplementary Table S1. Detailed search strategy in PubMed (it was adapted to each database). [file 13643_2023_2230_MOESM1_ESM.docx]

**Supplementary 1.** Detailed search strategy in PubMed (it was adapted to each database)

| #1 | "Psychometrics"[Mesh] OR ("Validation Studies as Topic"[Mesh] OR "Validation Studies"[Publication Type]) OR "Reproducibility of Results"[Mesh] OR "Exploratory Factor Analysis"[All Fields] OR "Confirmatory factor analysis"[All Fields] OR "Reliability"[All Fields] OR "Intra-class correlation coefficient" OR "Test-retest"[All Fields] OR "Inter-rater"[All Fields] OR "Intra-rater"[All Fields] OR "Internal Consistency"[All Fields] OR ("Validity"[All Fields] AND "Content Validity"[All Fields] OR "Face Validity"[All Fields] OR "Internal Validity"[All Fields] OR "Convergent Validity"[All Fields] OR "External Validity"[All Fields]) OR “Construct Validity” OR “Dimensionality Factor Structure” OR “Sensitivity to Change” OR “Responsiveness” OR “Sensibility” OR “Specificity” |
| --- | --- |
| #2 | (Attitude* AND Toward* AND Mental* AND Ill*) OR “CAMI” |
| #3 | #1 AND #2 |
